# Design and experimental validation of a soft pneumatic robotic device for preterm infant skin-to-skin tactile therapy

**DOI:** 10.3389/frobt.2026.1839026

**Published:** 2026-05-28

**Authors:** Yarilenka Benites-Mozo, Franco Daiji Huemura Okumura, Mahdi Tavakoli, Emir A. Vela

**Affiliations:** 1 Department of Mechanical Engineering, Universidad de Ingenieria y Tecnologia—UTEC, Lima, Peru; 2 Department of Electrical and Computer Engineering, University of Alberta, Edmonton, AB, Canada

**Keywords:** biomedical robotics, closed-loop control, neonatal tactile stimulation, pneumatic actuators, soft robotics, soft wearable devices

## Abstract

Skin-to-skin tactile stimulation plays a critical role in the neurodevelopment of preterm infants, contributing to improved physiological stability, sensory integration, and caregiver bonding. However, the delivery of consistent tactile therapy in neonatal intensive care units is often limited by the availability of trained personnel and the inherent variability of manual application. This creates a need for assistive technologies capable of reproducing clinically relevant tactile stimuli in a controlled and repeatable manner. This work presents a soft pneumatic robotic system designed to replicate clinically inspired tactile stimulation for neonatal therapy. The proposed approach integrates experimental characterization of manual tactile interactions, pneumatic system modeling, and closed-loop control. Force measurements obtained from a neonatology specialist were used to define clinically grounded stimulation levels, with mean values of 0.594 N for light stimulation and 1.267 N for moderate stimulation. These values were mapped to actuator pressure through experimentally identified linear relationships between force, pressure, and electrical current, enabling the definition of therapeutic pressure references. A 3 × 3 matrix of textile pneumatic actuators was integrated with an electro-pneumatic actuation system, pressure sensing, and current monitoring to implement the proposed therapy platform. Two control strategies were evaluated: a baseline pressure deadband controller and a refined deadband controller incorporating actuation tuning and current-based protection. Experimental results demonstrated that the refined controller increased the time within the therapeutic pressure band from 30.8% to 76.1%, while reducing mean pressure error from 0.68 kPa to 0.15 kPa and RMS error from 0.92 kPa to 0.38 kPa. Robustness tests under varying mechanical interface conditions showed stable and consistent pressure regulation performance. These results demonstrate the feasibility of translating clinically derived tactile stimulation into controlled pneumatic actuation using a soft robotic platform. By combining clinically grounded references, pressure-based closed-loop control, and safety-oriented current monitoring, the proposed system provides a reproducible and safe preclinical platform for neonatal tactile therapy and supports the development of assistive soft robotic technologies for clinically representative neonatal care environments.

## 1 Introduction

Preterm birth remains a major global health challenge, with approximately 10% of infants worldwide born before 37 weeks of gestation (World Health Organization, 2023). In Peru, recent national health reports indicate that premature births represent a growing concern within neonatal intensive care units (NICUs), where resource limitations and high patient demand place additional strain on clinical staff (Ministerio de Salud del Perú 2022; Peruano, 2023). Premature infants admitted to NICUs are exposed to multiple stressors, including excessive noise, continuous lighting, frequent medical interventions, and prolonged separation from caregivers. These environmental and procedural factors have been shown to negatively affect both short and long-term neurodevelopmental outcomes (Newnham et al., 2009; Santos et al., 2015; Smith, 2011).

Sustained exposure to stress during the neonatal period has been associated with physiological instability, altered sleep–wake cycles, feeding difficulties, and increased pain sensitivity in the short term, as well as cognitive, behavioral, and emotional impairments later in life (Brummelte, 2012; Doesburg, 2013). Consequently, non-pharmacological interventions aimed at stress reduction have become a priority in neonatal care. Among these, skin-to-skin therapy, also referred to as kangaroo care or therapeutic touch, has consistently demonstrated positive effects on autonomic regulation, pain reduction, and neurodevelopmental stability, even in medically complex or intubated preterm infants (Bedetti, 2023; Carbasse, 2013; Campbell-Yeo et al., 2022). Human perception of such tactile interactions arises from the integration of cutaneous and kinesthetic sensing mechanisms that encode force, pressure, and motion during physical contact (Tavakoli, 2014).

Skin-to-skin therapy encompasses several modalities, including breastfeeding, kangaroo positioning, and manual massage performed by trained healthcare professionals. In particular, skin-to-skin massage therapy involves rhythmic, gentle tactile stimulation applied to the infant’s abdomen, chest, and extremities, following predefined trajectories and intensities (Torres, 2023; Field, 2018). Although this therapy has proven clinical benefits, its routine implementation in NICUs, especially in low and middle income countries, is limited by a shortage of specialized personnel, time constraints, and infrastructural challenges (de Salud, 2022; Spence et al., 2023). These limitations motivate the exploration of technological solutions capable of supporting or partially automating therapeutic tactile stimulation in a safe and controlled manner.

Robotic and mechatronic systems have been proposed as alternatives to assist neonatal care, primarily focusing on environmental regulation and multisensory stimulation. Devices such as Calmer and BabyBee have demonstrated the feasibility of using robotic platforms to reproduce maternal breathing patterns or gentle rocking motions to reduce neonatal stress (Holsti, 2019; Kommers, 2018). However, these systems do not replicate the localized, distributed, and pressure-controlled tactile interaction characteristic of skin-to-skin massage therapy, which remains one of the most effective non-pharmacological interventions for stress reduction (Campbell-Yeo et al., 2022). As a result, the development of tactile stimulation patterns observed in clinical practice remains an open research challenge.

Recent advances in soft robotics provide a promising framework for addressing this gap. Soft pneumatic actuators fabricated from compliant materials offer inherent safety, adaptability to complex surfaces, and mechanical properties closer to biological tissues than rigid robotic systems (Schmitt, 2018; Zhang et al., 2020). Their intrinsic compliance reduces the risk of excessive mechanical interaction and makes them particularly suitable for applications involving direct human contact. Prior studies have explored soft robotic massage devices for adult therapeutic applications, demonstrating comparable outcomes to manual therapy in terms of pain reduction, muscle relaxation, and perceived comfort (Kerautret et al., 2023; Zhu et al., 2023).

In the neonatal context, a previous study by the same research group (Huemura Okumura et al., 2026) introduced a compact soft pneumatic actuator array based on a nylon–TPU textile platform. That work demonstrated the feasibility of the device through a passive fluidic sequencing architecture, enabling predefined actuation patterns as a proof of concept for safe, massage-like tactile stimulation in preterm infants. However, the system was limited to passive actuation and did not incorporate quantitative clinical references, active control strategies, or programmable stimulation patterns. In particular, no direct relationship was established between the robotic actuation and clinically measured force inputs.

The present manuscript builds upon this foundation by advancing the system toward a fully operational and controllable platform. It incorporates force measurements derived from specialist-applied neonatal massage, thereby establishing clinically grounded reference values for stimulation intensity. Furthermore, it introduces a control framework that enables programmable spatiotemporal activation patterns, including linear, circular, and diagonal sequences. As a result, the contribution of this work lies not only in force characterization, but in the integration of clinically informed actuation targets with an actively controlled multi-actuator system capable of reproducing structured tactile stimulation patterns.

Despite these advances, an important limitation in current robotic tactile stimulation systems is the lack of direct integration between clinical measurements and robotic actuation design. Many existing devices define stimulation parameters based primarily on actuator capabilities rather than on experimentally measured therapeutic forces applied by clinicians. Consequently, the resulting robotic stimuli may not accurately reproduce the mechanical characteristics of clinically validated tactile therapies. Addressing this gap requires a methodology capable of translating clinically observed tactile interactions into quantifiable mechanical parameters that can be reproduced by robotic systems.

In this context, the hypothesis guiding this work is that a soft pneumatic robotic system designed using experimentally characterized tactile stimuli applied by a neonatology specialist can safely approximate the force patterns, pressures, and activation sequences involved in skin-to-skin massage therapy for premature infants. To investigate this hypothesis, this study proposes a system-level approach that integrates clinical measurement, pneumatic actuator characterization, and embedded control design within a unified experimental framework.

First, tactile forces and movement trajectories generated during manual therapy were experimentally recorded using a dedicated measurement platform. The resulting signals were statistically analyzed to determine representative force intervals and temporal characteristics of the stimulation patterns applied by the specialist. Second, an electro–pneumatic actuation platform was developed to reproduce controlled pressure inputs corresponding to these clinically derived force ranges. Third, a wearable soft robotic device consisting of a 3 × 3 matrix of fabric-based pneumatic actuators was designed to deliver spatially distributed tactile stimulation across the target region. Finally, closed-loop control strategies were implemented and experimentally evaluated to regulate actuator pressure within the therapeutic intervals while maintaining safe electrical operating conditions.

This study makes three main contributions to biomedical soft robotics. First, it proposes a methodology to translate clinically measured tactile forces into pneumatic control references for robotic actuation. Second, it develops a soft pneumatic actuator matrix capable of reproducing spatial stimulation patterns derived from clinical observations. Third, it evaluates safety-oriented control strategies to maintain stimulation within predefined therapeutic limits. Experimental validation using a neonatal mannequin demonstrates the feasibility of reproducing clinically inspired tactile patterns while ensuring controlled mechanical output and stable pneumatic operation.

By grounding the system design in experimental clinical data and prioritizing safety through compliant actuation, sensing, and control, this work advances assistive soft robotic technologies for neonatal care. Such systems may support NICU environments where clinical resources are limited and consistent delivery of tactile therapy is difficult to maintain. More broadly, this approach demonstrates how soft robotics can integrate clinical knowledge to enable reproducible and quantitatively controlled therapeutic stimulation.

## 2 Methodology

**FIGURE 1:**
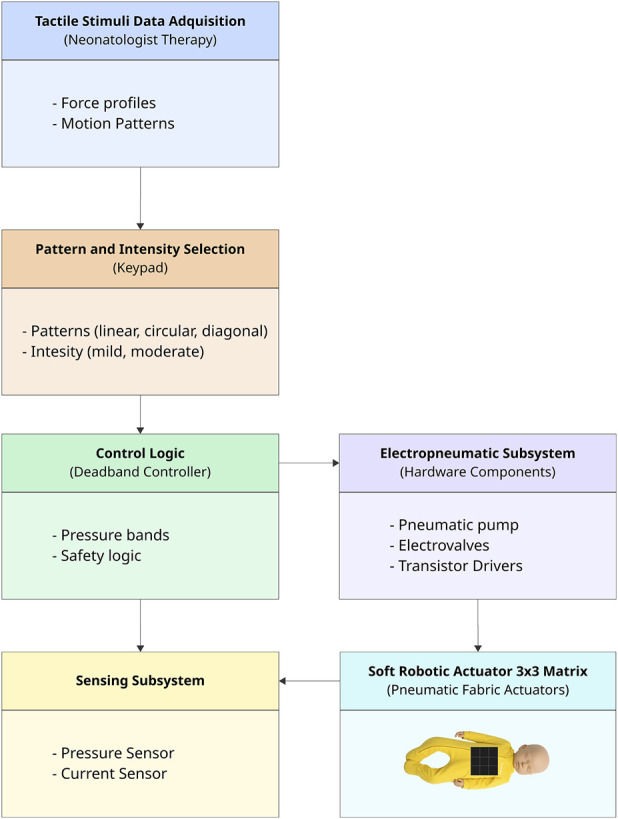
General architecture of the proposed soft robotic system.

Figure 1 presents the overall architecture of the proposed soft robotic system, designed to reproduce clinically inspired tactile stimulation for neonatal therapy. The system follows a structured pipeline that links clinical observation, signal processing, control design, and pneumatic actuation.

In the first stage, tactile stimuli applied by a neonatology specialist were recorded using an experimental measurement platform to capture force levels and motion patterns representative of clinical practice. These data were processed to extract reference parameters, including stimulation intensity and temporal characteristics, which serve as inputs for the control system.

Based on this characterization, predefined stimulation patterns and intensity levels were defined and selected through a user interface. These commands are executed by an embedded control layer implementing a deadband-based strategy with safety constraints.

The control signals drive an electro-pneumatic subsystem composed of a diaphragm pump, solenoid valves, and driver circuits, which regulate airflow to a 3×3 matrix of fabric-based pneumatic actuators. This matrix constitutes the physical interface and is mounted on a neonatal mannequin to reproduce controlled contact conditions.

A sensing subsystem provides real-time feedback through pressure and current measurements. The pressure sensor monitors the pneumatic state of the actuator array, while the current sensor supervises the electrical load of the pump. These signals close the control loop, enabling regulation of the applied stimulus within predefined therapeutic limits while ensuring safe operation.

### 2.1 Experimental characterization of the tactile stimulus applied by the specialist

The tactile stimuli applied during neonatal therapy were experimentally recorded to obtain clinically grounded reference parameters. The experiments were conducted with an experienced neonatology clinician who performed manual tactile interactions following standard medical practice.

The recorded data were used to characterize both the applied force and the temporal structure of the stimulation. These parameters were subsequently used to define reference values for the control system design.

#### 2.1.1 Characterization of applied force

To quantify the tactile stimuli, a dedicated measurement platform was used to capture the forces applied by the specialist under controlled laboratory conditions. The experiments were conducted on the platform while the doctor performed the movements based on his experience with real infants, to ensure repeatability and safety.

##### 2.1.1.1 Mechanical and electronic design of the measurement platform

**FIGURE 2:**
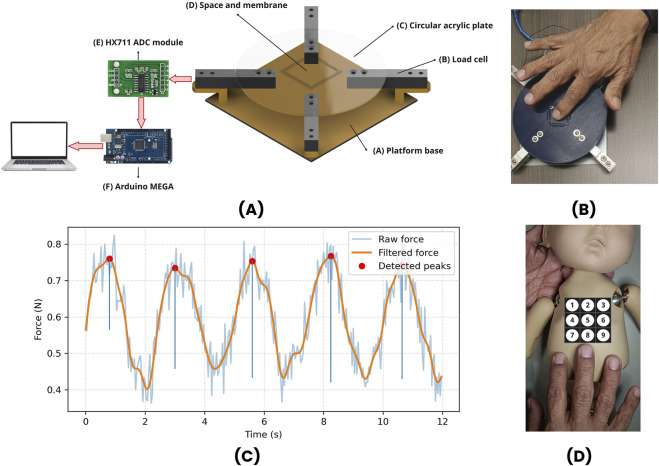
Extraction and translation of tactile stimulation characteristics **(A)** shows the measurement platform **(B)** shows neonatology specialist applying static pressures at the center of the measurement platform **(C)** shows temporal signal filtering and peak detection for period estimation. **(D)** Shows mapping of motion patterns to the 3 × 3 pneumatic actuator matrix.

The measurement platform (Figure 2A) was designed to reproduce the mechanical conditions of finger–skin contact during therapy. The structural base was fabricated in PLA using fused deposition modeling (FDM), providing rigidity and dimensional stability. It has a square geometry of 140 mm × 140 mm and integrates four aluminum load cells uniformly distributed beneath the contact surface to mitigate errors due to off-center loading during manual interaction.

A rigid acrylic plate was mounted above the load cells to ensure uniform force transmission. At its center, a cavity of 20 mm × 30 mm × 1 mm houses a thin Ecoflex^TM^ 00-35 FAST silicone membrane, which acts as a compliant, skin-mimicking interface. The contact area dimensions were selected based on anthropometric measurements of the distal phalanx with a length between 16 and 20 mm and between width 14–18 mm (Cutkosky, 1989; Napier, 1993), ensuring consistent and natural interaction.

Ecoflex^TM^ 00-35 FAST was selected due to its widespread use in soft robotics and its mechanical similarity to soft biological tissues. It exhibits a Shore hardness of 00-35 and a Young’s modulus of approximately 125–170 kPa, depending on curing conditions (Smooth-On Inc, 2023; Polygerinos et al., 2015). These properties enable deformation under low force levels while maintaining structural integrity under repeated loading.

Each load cell (1 kg nominal capacity) was configured in a full Wheatstone bridge and interfaced with a 24-bit HX711 analog-to-digital converter, enabling accurate measurement of low-magnitude forces. The total applied force was computed as the sum of the four synchronized load cell outputs. Although off-center loading results in an uneven distribution of force among the sensors, the rigid coupling provided by the acrylic plate ensures that the system satisfies static equilibrium, such that the sum of the reaction forces corresponds to the total applied force regardless of load position. The use of four load cells further improves stability and reduces sensitivity to torque and off-axis loading. Any minor coupling effects between sensors are implicitly accounted for through the calibration procedure.

##### 2.1.1.2 Acquisition of experimental data

The neonatology specialist applied controlled static pressures on the measurement platform at two intensity levels (light and moderate), following standard clinical practice (Figure 2B). For each level, five repetitions of 12 s were recorded. In all measurements, the pressure was applied using a single-point contact.

Prior to data acquisition, the platform was calibrated through a taring and scaling procedure to ensure accurate force measurement. The total raw signal was computed as the sum of the four load cell outputs, as defined in Equation 1:

$$H_{\mathrm{tot}}=\overset{4}{\underset{i=1}{\sum }}H_{i}$$ (1)

The offset was estimated from baseline measurements of the unloaded platform and removed from the signal. A scale factor was then determined using a known reference mass, allowing conversion of raw sensor values into physical units.

The applied force was computed using the calibrated signal, as expressed in Equation 2:

$$F=\frac{H_{\mathrm{tot}}-offset}{scale}\cdot g$$ (2)

where $g=9.78\;{\text{m/s}}^{2}$ corresponds to the local gravitational acceleration. This calibration procedure ensures accurate and repeatable estimation of the forces applied during tactile interaction.

##### 2.1.1.3 Data processing to obtain reference force levels

The force signals acquired during the experimental sessions were processed to extract representative temporal and amplitude features associated with tactile stimulation. Given the low-frequency characteristics of the recorded tactile interactions in this study, the processing focused on preserving the relevant biomechanical components while attenuating high-frequency noise and measurement artifacts.

The signals were sampled at a frequency of $f_{s}=25$ Hz. This sampling rate is sufficient to capture the temporal characteristics of human-applied tactile interaction, which are dominated by low-frequency components. Previous studies have shown that caregiving and therapeutic touch concentrate most of their spectral energy below 5 Hz, with relevant biomechanical content typically under 2 Hz (Culbertson et al., 2018; Jones and Lederman, 2006). Therefore, the selected sampling frequency provides adequate temporal resolution while avoiding unnecessary data redundancy.

A fourth-order low-pass Butterworth filter was applied to attenuate high-frequency noise while preserving the relevant biomechanical content of the signal. This filter type was selected due to its maximally flat passband response, which minimizes amplitude distortion. To avoid phase distortion and temporal displacement of force peaks, zero-phase forward–backward filtering was implemented.

Representative tactile events were identified using prominence-based peak detection. Unlike simple amplitude thresholding, this method evaluates each peak relative to its local baseline, providing robustness against slow drifts and residual noise. A minimum prominence threshold and inter-peak distance were empirically selected to ensure consistent detection across trials. Figure 2C illustrates the effect of filtering and peak detection on the measured force signal.

The detected peaks correspond to the maximum force values reached during each stimulation cycle and were used to characterize the tactile stimulus. Let $F_{\mathrm{peaks}}$ denote the set of force values extracted at the detected peak locations. The representative force level was computed as defined in Equation 3:

$$\mu_{F}=\frac{1}{N}\overset{N}{\underset{i=1}{\sum }}F_{\mathrm{peaks,i}}$$ (3)

where N is the total number of detected stimulation events. The variability of the tactile stimulus was quantified using the standard deviation of the peak forces, providing a measure of consistency across repetitions.

#### 2.1.2 Characterization of movement patterns and inflation duration

To replicate the dynamic tactile stimulation applied by the specialist, a computer vision–based kinematic analysis was performed to extract temporal and directional characteristics of manual interaction and translate them into actuation timing and activation sequences.

The robotic system does not reproduce continuous sliding motion mechanically. Instead, it generates the perception of dynamic caress using a 3 × 3 matrix of individually controlled pneumatic actuators (Figure 2D). Each actuator produces localized static pressure, while dynamic motion is approximated through the spatiotemporal sequencing of adjacent actuators.

This approach constitutes a discrete spatiotemporal approximation of continuous tactile displacement, where coordinated activation produces a distributed tactile flow across the surface.

#### 2.1.3 Experimental setup and video acquisition

Videos were recorded using a neonatal mannequin (StandInBaby®) representing a full-term newborn. The mannequin provides anatomically realistic dimensions and compliant surface properties suitable for controlled experimentation.

Recordings were performed using a smartphone camera at 30 fps under consistent lighting and positioning conditions. The specialist executed three representative tactile patterns corresponding to common stimulation strategies: linear, circular, and diagonal.

These patterns were selected to approximate fundamental motion primitives observed in neonatal tactile stimulation practices. However, existing literature typically characterizes these interventions in terms of pressure, duration, and frequency, without explicitly defining or comparing the directional trajectories of tactile motion (e.g., linear, circular, or diagonal strokes) (Zaslavski, 2022; Ferreira and Bergamasco, 2010; Reco and Soares-Marangoni, 2024).

Given this lack of standardized descriptions of motion direction, the selected patterns were defined as a minimal and representative set of spatiotemporal primitives to enable controlled reproduction and systematic evaluation of tactile stimulation using the discrete 3 × 3 actuator array. In this context, linear patterns approximate directional stroking, circular patterns represent cyclic soothing motions, and diagonal patterns introduce directional variability across the stimulation surface.

#### 2.1.4 Kinematic analysis and temporal parameter extraction

Video processing was implemented in Python using OpenCV for frame acquisition (Bradski, 2000) and MediaPipe for hand landmark detection (Lugaresi et al., 2019). The coordinates of the middle fingertip (landmark 12) were extracted for each frame, generating time series x(t) and y(t) .

Since the objective was to extract temporal characteristics, spatial calibration was not required. The vertical displacement was normalized as defined in Equation 4 to remove offsets while preserving motion dynamics:

$$y_{\mathrm{norm}}\left(t\right)=y\left(t\right)-\min\left(y\right)$$ (4)

Noise and high-frequency fluctuations were attenuated using a low-pass Butterworth filter with zero-phase implementation. Repetitive tactile strokes were identified using prominence-based peak detection, and the mean oscillation period was computed using Equation 5:

$$T_{\mathrm{mean}}=\frac{1}{N-1}\overset{N-1}{\underset{i=1}{\sum }}\left(t_{i+1}-t_{i}\right)$$ (5)

The observed therapist motion consists of a periodic up–down interaction, where each cycle corresponds to a complete traversal of the contact region. In the robotic implementation, this continuous motion is discretized onto the 3 × 3 actuator array, where each directional pattern is represented by a sequence of three consecutive actuator positions along the trajectory.

To preserve the temporal characteristics of the original motion, the actuator inflation time was defined as a fraction of the oscillation period, as shown in Equation 6:

$$T_{\mathrm{inflation}}=\frac{T_{\mathrm{mean}}}{3}$$ (6)

This definition ensures that the sequential activation of the three actuator positions reproduces the duration of a complete tactile cycle observed in the therapist’s motion.

Although the therapist applied the interaction using three fingers simultaneously, their movement was coordinated, resulting in a localized distributed contact pattern. For this reason, the trajectory of the middle fingertip was used as a representative reference to define the directional progression of the tactile patterns and to map this progression onto the corresponding activation sequence of the actuator matrix. This simplification enables consistent extraction of motion features while preserving the temporal structure of the interaction.

### 2.2 Design and implementation of the soft robotic device

**FIGURE 3:**
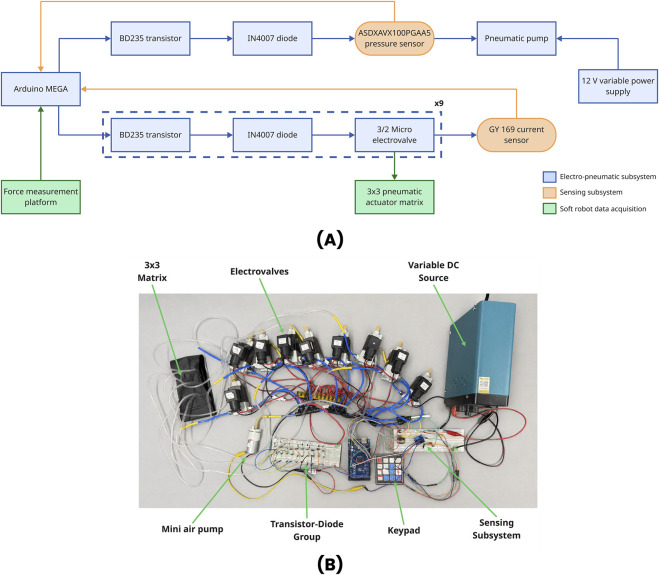
Design and implementation of the soft robotic system. **(A)** Conceptual hardware architecture including electro-pneumatic actuation, sensing, and data acquisition subsystem. **(B)** Shows complete implemented soft robotic device.

The soft robotic system was designed to reproduce clinically inspired tactile stimulation through a compact and integrated electro-pneumatic platform. The implementation combines a textile-based 3 × 3 pneumatic actuator matrix, a centralized air supply with distributed control, and sensing for monitoring system behavior. The overall design, including actuator fabrication, hardware architecture, and sensing integration, is illustrated in Figure 3.

#### 2.2.1 Fabrication of the 3 × 3 soft actuator matrix

The actuator matrix was fabricated using TPU-coated nylon fabric due to its flexibility, mechanical robustness, and airtightness, which are essential for textile pneumatic actuators (Coram et al., 2024; Huemura Okumura et al., 2026). Thermal bonding via heat sealing was selected as it provides consistent airtight seams while preserving material compliance (Park et al., 2019; Goshtasbi et al., 2025).

A working area of 6 cm × 9 cm was defined based on neonatal anthropometry, ensuring coverage of both thoracic and abdominal regions (Meldere et al., 2015; Shoji et al., 2022). This area was divided into nine chambers of 2 cm × 3 cm arranged in a 3 × 3 matrix.

**FIGURE 4:**
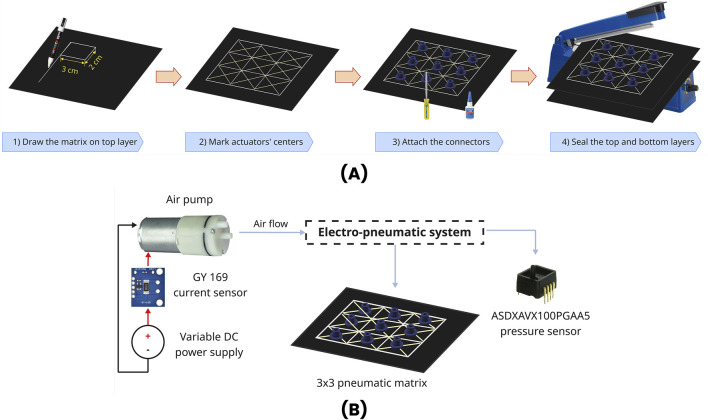
Fabrication process of the soft robotic device **(A)** shows step-by-step fabrication process of the 3 × 3 textile pneumatic actuator matrix: (1) matrix marking on TPU fabric; (2) marking chamber centers; (3) insertion of PLA connectors and adhesive sealing; (4) heat-sealing of top and bottom fabric layers. **(B)** Sensing subsystem components.

Each chamber was connected to the pneumatic system through a custom 3D-printed PLA connector inserted at its center (Figure 4A). The connectors enable secure tubing attachment and airtight interfacing. After insertion, adhesive sealing was applied, and the actuator layers were thermally bonded using a heat sealer to define internal chambers and external boundaries.

The resulting structure was visually inspected and tested under low pressure to ensure airtightness and uniform fabrication.

#### 2.2.2 Hardware architecture of the soft robotic system

The system integrates three main subsystems (Figure 3A): (1) electro-pneumatic actuation, (2) sensing, and (3) data acquisition.

Airflow generation was provided by a miniature diaphragm pump (DC motor type 370, 6 V), capable of delivering approximately 2 L/min and pressures up to 300 mmHg, ensuring sufficient dynamic response for actuator inflation.

Air distribution was achieved using independent 3/2 normally closed solenoid valves (12 VDC), enabling selective pressurization of each actuator chamber and programmable spatial stimulation patterns.

The pump airflow was regulated using PWM control implemented via a BD235 NPN transistor switching stage driven by an Arduino Mega 2560 microcontroller. The Arduino platform was selected due to its large number of digital I/O pins and PWM channels, enabling independent valve control and real-time system coordination.

Component selection prioritized compatibility with low-pressure biomedical applications, electrical safety, and system reliability.

#### 2.2.3 Sensing subsystem

The sensing subsystem enables simultaneous monitoring of pneumatic and electrical variables (Figure 4B). Pneumatic pressure was measured using a Honeywell ASDX-series pressure sensor (model ASDXAVX100PGAA5), providing a ratiometric analog output over a calibrated pressure range.

The conversion from sensor voltage to pressure was computed as defined in Equation 7:

$$P=\frac{V_{\mathrm{out}}-V_{\min}}{V_{\max}-V_{\min}}P_{FS}$$ (7)

In parallel, the electrical current of the pump was measured using a GY-169 Hall-effect current sensor, providing an indirect indicator of mechanical load and airflow demand.

Because the current sensor required calibration, an experimental procedure was performed using reference measurements and linear regression, as expressed in Equation 8:

I=a⋅ADC+b (8)

To reduce measurement noise, filtering strategies were applied, including averaging and exponential smoothing.

#### 2.2.4 Pressure and current data acquisition

To define reference control values, the relationship between pneumatic pressure and applied force was experimentally characterized using a neonatal mannequin as a surrogate for body geometry.

A clinically acceptable interface condition was established by adjusting a diaper on the mannequin under physician guidance. To reproduce this condition, PLA-printed spacers of varying thickness were evaluated, resulting in a selected clearance of 25 mm.

A single actuator (3 cm × 2 cm) was used for characterization. The actuator was mounted on the measurement platform, and pneumatic pressure, applied force, and pump current were recorded simultaneously.

The actuator was driven using PWM signals in discrete increments, allowing stable measurements at each operating point. The resulting dataset defines the mapping described in Equation 9:

$$PWM\to \left(P,F,I\right)$$ (9)

##### 2.2.4.1 Signal processing

To mitigate noise introduced by pneumatic actuation and pump operation, filtering was applied to both current and pressure signals. Current measurements were processed using trimmed averaging, followed by exponential smoothing, as defined in Equation 10:

$$I_{f}\left(k\right)=\alpha I_{m}\left(k\right)+\left(1-\alpha \right)I_{f}\left(k-1\right)$$ (10)

Similarly, pressure measurements were filtered using a first-order exponential filter, as shown in Equation 11:

$$P_{f}\left(k\right)=0.7P_{f}\left(k-1\right)+0.3P_{m}\left(k\right)$$ (11)

### 2.3 Closed-loop control implementation

The device operates through pneumatic inflation of a matrix of soft actuators, regulated using pressure feedback to ensure safe and repeatable tactile stimulation. Pneumatic systems inherently exhibit nonlinear behavior due to air compressibility, material elasticity, and flow restrictions, leading to hysteresis and transient delays. Additionally, the short actuation time limits the effectiveness of continuous controllers such as PID, which may introduce oscillations under fast transient conditions.

**FIGURE 5:**
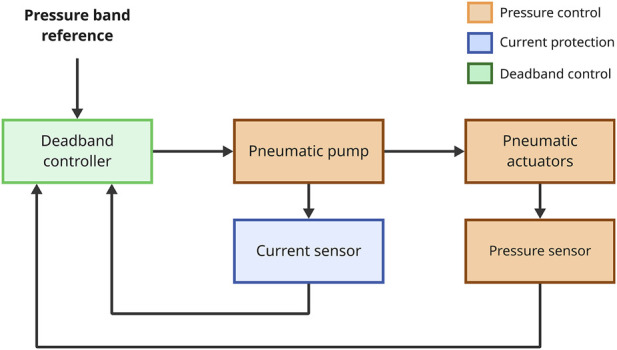
Closed-loop control architecture showing the deadband pressure regulation (orange) and the current-based protection layer (blue).

To address these constraints, a deadband control strategy was adopted. This approach maintains the actuator pressure within a predefined therapeutic interval, reducing switching frequency and improving robustness under discrete pneumatic actuation. Two configurations were implemented: (1) pressure-based deadband control and (2) an extended version incorporating electrical current protection. The overall control architecture is illustrated in Figure 5, where the pressure regulation loop (orange) and the current protection layer (blue) are shown.

#### 2.3.1 Deadband pressure controller

The control objective is to regulate the pressure difference ΔP within therapeutic bounds $\left[P_{\min},P_{\max}\right]$ , derived from the experimental characterization. As shown in Figure 5 (orange block), the controller operates on pressure feedback obtained from the pneumatic line.

Pressure is estimated from the sensor voltage as defined in Equation 12

$$P=\left(V-0.5\right)\times 25$$ (12)

and corrected using an offset measured during initialization, as expressed in Equation 13

$$\Delta P=P-P_{\mathrm{offset}}$$ (13)

The control law is defined as shown in Equation 14

$$u\left(t\right)=\left\{\begin{array}{cc} PWM_{on} & \text{if }\Delta P<P_{\min}\\ 0 & \text{if }\Delta P>P_{\max}\\ u\left(t-1\right) & \text{otherwise} \end{array}\right.$$ (14)

where $PWM_{on}$ is a fixed duty cycle. In this implementation, $PWM_{on}=225$ was selected empirically, based on the observed dynamic response of the pneumatic system, to provide sufficient actuation to reach the therapeutic pressure range within the available stimulation time.

In this initial controller, the same PWM value was used for both light and moderate stimulation levels. Under this configuration, the pressure bounds define the effective stimulation intensity, while the actuation signal drives the system toward the desired range. However, due to the fast and nonlinear response of the pneumatic actuator, this approach may lead to relatively aggressive inflation dynamics, particularly at lower force levels.

The inclusion of u(t−1) introduces hysteresis, preventing rapid switching near the threshold boundaries. This structure enables stable regulation within the therapeutic range while minimizing actuation effort.

The selection of a deadband-based control strategy is motivated by the dynamic characteristics of the pneumatic system and the temporal constraints of the application. The stimulation time per actuator, defined from clinical characterization, is $T_{r}=1.739\;s$ , which requires rapid convergence to the target pressure range. Under these conditions, continuous control strategies such as PID introduce additional settling time and may lead to overshoot due to the nonlinear and saturating behavior of the pneumatic actuator. The deadband approach enables immediate actuation and bounded behavior, making it more suitable for this application.

#### 2.3.2 Deadband controller with current protection

To address the limitations observed in the initial controller, a refined actuation strategy was implemented. In this configuration, different PWM values were used for each stimulation level, with PWM=110 for light stimulation and PWM=255 for moderate stimulation. This adjustment reduces the aggressiveness of the response at lower force levels while maintaining fast convergence for higher pressures.

To enhance operational safety, current monitoring was incorporated as a secondary protection layer. As shown in Figure 5 (blue block), the protection mechanism supervises the pump operation independently of the pressure loop.

The pump current is estimated from the analog measurement using a calibrated linear model, as defined in Equation 15

I=a⋅ADC+b (15)

A protection threshold $I_{\mathrm{fuse}}$ is defined, and the pump is disabled if the condition in Equation 16 is satisfied

$$I>I_{\mathrm{fuse}}$$ (16)

In this implementation, the protection threshold was set to $I_{\mathrm{fuse}}=2\;A$ , and a delay of 400 ms was introduced to avoid triggering during startup transients. These values were determined experimentally based on repeated trials under representative operating conditions, balancing fast pressure convergence, reduced overshoot, and safe electrical operation.

This mechanism acts as a software-based fuse, preventing damage under abnormal conditions while preserving pressure regulation. The combination of adaptive PWM levels and current protection improves stability and safety while maintaining responsiveness within the limited stimulation time.

### 2.4 Experimental protocol for controller evaluation

Experiments were conducted using the neonatal mannequin described previously. The actuator matrix was attached using hook-and-loop fasteners to ensure repeatable positioning while preserving compliant contact.

The interface condition was defined in terms of the effective separation between the actuator and the neonatal surface, rather than the physical thickness of the diaper alone. During experimental setup, the diaper was applied to the neonatal mannequin following standard clinical practice, ensuring a secure fit without excessive compression or skin marking. This condition reflects a practical balance between stability and safety in neonatal care, where the interface is determined by the combined effect of diaper bulk, fastening, and mounting configuration.

To translate this clinically guided condition into a reproducible experimental parameter, the effective actuator–surface separation distance was measured under this configuration. Custom PLA elements were then used to reproduce and standardize this separation, enabling controlled and repeatable actuator–surface interaction conditions. PLA spacers were selected as rigid and dimensionally stable elements, allowing precise control of the separation distance while minimizing the influence of material deformation. This approach isolates geometric spacing as the primary variable under study, rather than attempting to replicate the mechanical compliance of the diaper.

Based on this characterization, a reference separation distance of 25 mm was selected, while additional configurations (17.5 mm and 50 mm) were evaluated to assess robustness to variations in interface conditions.

Three stimulation patterns (linear, circular, and diagonal) were evaluated, each corresponding to predefined activation sequences of the 3×3 actuator matrix. The stimulation time per pouch was defined from clinical characterization as $T_{r}=1.739\;s$ . The duration of each stimulation pattern is given by Equation 17:

$$T_{\mathrm{pattern}}=N_{p}\;T_{r}$$ (17)

where $N_{p}$ is the number of actuators activated per pattern. A complete therapy cycle consists of the sequential execution of the three patterns.

#### 2.4.1 Performance metrics

System performance was evaluated using three complementary metrics: percentage of time within the therapeutic band, mean pressure error, and root-mean-square (RMS) error.

The percentage of time within the therapeutic band quantifies the ability of the controller to maintain the pressure within clinically defined limits. The mean error provides a measure of steady-state deviation, while the RMS error captures both steady-state and transient deviations.

Variability across repeated trials was assessed using the standard deviation of the measured signals, providing a direct and robust characterization of repeatability under dynamic operating conditions.

#### 2.4.2 Statistical analysis

For each experimental condition, results were computed over multiple repetitions and summarized using mean and standard deviation values. These statistical descriptors provide a consistent representation of central tendency and variability across trials.

Given the dynamic nature of the system, characterized by sequential actuator activation and transient responses, variability is more appropriately captured using standard deviation and RMS-based metrics rather than normalized measures.

## 3 Results

This section presents the experimental evaluation of the proposed system, focusing on the characterization of clinically derived stimulation levels, the validation of the force, pressure and current relationship, and the performance of the control strategies under representative operating conditions. The results aim to assess the ability of the system to reproduce clinically inspired tactile stimuli while ensuring stable and safe operation within predefined therapeutic bounds.

First, force and motion characteristics are quantified to define clinically grounded reference parameters. These are mapped to the pneumatic domain through actuator characterization, establishing relationships between force, pressure, and current. Finally, the control strategies are evaluated based on their ability to regulate pressure within therapeutic bounds under dynamic conditions.

Together, these results demonstrate that clinically defined tactile stimulation can be consistently reproduced through controlled pneumatic actuation using the proposed system.

### 3.1 Experimental characterization of the tactile stimulus by the specialist

The processed force signals, obtained after filtering and peak detection, were analyzed to define quantitative force intervals for each stimulation level. These intervals establish clinically grounded reference values for the subsequent control design.

#### 3.1.1 Force characterization of the tactile stimulus

**FIGURE 6:**
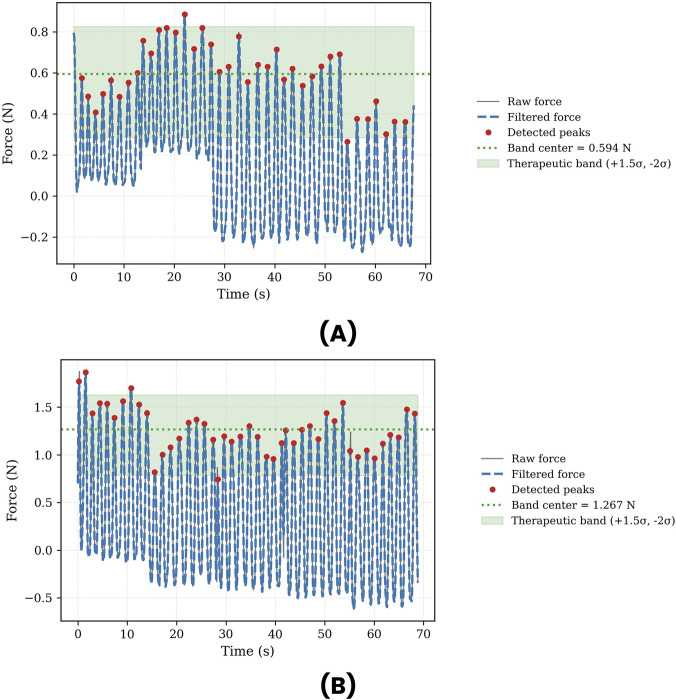
Processed force signal and detected peaks: **(A)** light tactile stimulation and **(B)** moderate tactile stimulation.

Figure 6A shows the filtered force signal and detected peaks for the light stimulation level. The signal exhibits a clear periodic structure corresponding to successive tactile interactions, while the Butterworth filter effectively attenuates measurement noise without distorting the force profile.

The statistical characterization yielded:

$$\begin{array}{ll} \mu_{\text{light}} & =0.594\;\text{N},\\ \sigma_{\text{light}} & =0.155\;\text{N} \end{array}$$

The corresponding therapeutic band was defined as:

$$\begin{array}{ll} F_{\text{lower}} & =0.285\;\text{N},\\ F_{\text{upper}} & =0.826\;\text{N} \end{array}$$

Most detected peaks lie within this interval, indicating consistent force application with controlled variability. The therapeutic band was defined asymmetrically to obtain a representative range of the measured interaction while reducing the influence of high-force outliers. A tighter margin was applied to the upper bound (μ+1.5σ) to limit higher-force deviations, whereas a wider margin was used for the lower bound (approximately μ−2σ ) to preserve the full range of typical interaction forces.

Figure 6B presents the results for the moderate stimulation level. The force amplitude increases relative to the light condition while preserving a similar temporal structure, indicating that the clinician modulates intensity without altering the interaction dynamics.

The statistical characterization yielded:

$$\begin{array}{ll} \mu_{\text{moderate}} & =1.267\;\text{N},\\ \sigma_{\text{moderate}} & =0.241\;\text{N} \end{array}$$

with a therapeutic band defined as:

$$\begin{array}{ll} F_{\text{lower}} & =0.786\;\text{N},\\ F_{\text{upper}} & =1.629\;\text{N} \end{array}$$

The majority of peaks remain within these bounds, demonstrating stable scaling of force between stimulation levels with predictable variability under the same band definition.

Although the mean force approximately doubles compared to the light condition, it remains within a low-force regime suitable for safe tactile interaction.

These results define clinically grounded force intervals that are subsequently mapped to pneumatic pressure and used to establish the control reference bands of the system.

#### 3.1.2 Characterization of movement patterns and inflation duration

**FIGURE 7:**
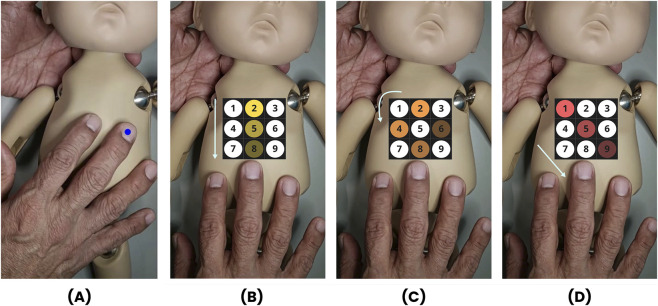
Extraction of motion patterns: **(A)** fingertip detection using MediaPipe and OpenCV, and resulting trajectories for **(B)** linear, **(C)** circular, and **(D)** diagonal patterns.

Video-based kinematic analysis (Figure 7A) revealed consistent oscillatory behavior across the three tactile patterns: linear, circular, and diagonal. The extracted fingertip trajectories (Figures 7B–D) capture the spatial progression of the applied stimulus.

**FIGURE 8:**
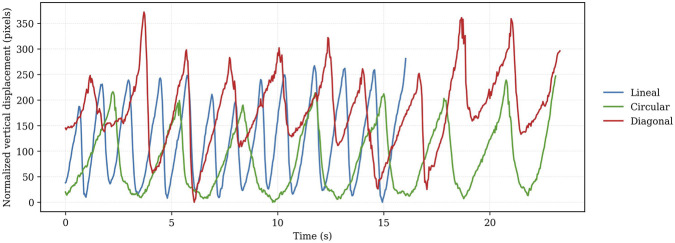
Normalized vertical displacement trajectories for the three tactile patterns.

The mean oscillation periods, obtained from peak detection of the normalized displacement signals (Figure 8), were 5.125 s (linear), 5.212 s (circular), and 5.314 s (diagonal), with an overall average of 5.217 s. The low dispersion across patterns indicates a consistent temporal cadence independent of trajectory geometry.

Since each trajectory traverses three actuator positions within the 3×3 matrix, the inflation time was defined as one-third of the oscillation period, resulting in $T_{r}=1.739$ s per actuator.

The extracted trajectories were mapped into discrete activation sequences of the actuator array:

- Linear: 1 → 4 → 7 → 2 → 5 → 8 → 3 → 6 → 9
- Circular: 2 → 4 → 8 → 6
- Diagonal: 7 → 4 → 8 → 1 → 5 → 9 → 2 → 6 → 3

These sequences represent a discretization of the continuous fingertip trajectories onto the spatial layout of the actuator array. The continuous motion is projected onto the nearest actuator positions along the path, generating ordered activation sequences that approximate the spatial progression of the stimulus across the surface.

The extracted trajectories may contain symmetric or repeated motion components. In such cases, the underlying pattern is represented using a single representative trajectory that captures the essential directional progression of the movement. This representation reduces redundancy while preserving the characteristic spatial structure of the interaction, enabling consistent mapping to the actuator array.

The resulting temporal and spatial parameters were then used to define the actuation timing and activation sequences implemented in the control system.

#### 3.1.3 Pneumatic characterization and reference pressure determination

The pneumatic actuation system was experimentally characterized to establish a direct relationship between clinically measured tactile forces, internal actuator pressure, and electrical operating conditions. This characterization enables the translation of clinically defined stimulation levels into physically realizable control references. By identifying consistent operating points and deriving simplified models relating force, pressure, and current, the system behavior can be represented properly for real-time control. The following results define these relationships and the corresponding pressure intervals used as reference bands for the deadband-based control strategy.

#### 3.1.4 Sensing subsystem implementation

The current sensing channel was calibrated to determine the relationship between the analog-to-digital converter (ADC) output and the actual pump current. A linear regression model fitted over measurements obtained under multiple PWM conditions resulted in Equation 18:

I=0.003338⋅ADC−0.026673 (18)

**FIGURE 9:**
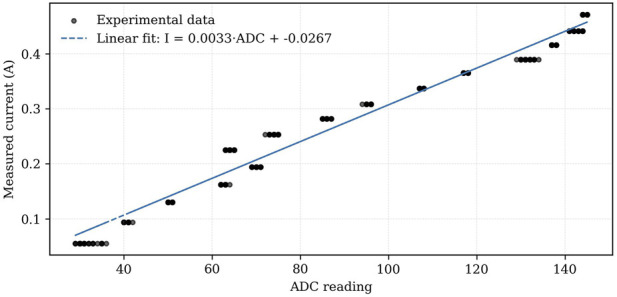
Experimental calibration of the GY-169 current sensor. Measured current as a function of ADC reading with least-squares linear regression fit.

Figure 9 shows the experimental data and fitted model. The data exhibit a strong linear trend across the operating range ( ≈0.05 –0.45 A), with no observable saturation or nonlinear distortion. Residual analysis yielded a mean error of 0.000 A, standard deviation of 0.0205 A, and maximum absolute error of 0.0414 A. The low dispersion (approximately 4%–5% of nominal current) confirms that a first-order model provides sufficient accuracy for real-time estimation.

Pressure measurements were obtained using the ASDXAVX100PGAA5 sensor, which provides a factory-calibrated ratiometric output with a total error band of ±2% full-scale span and a response time of approximately 1 ms. During operation, pressure signals exhibited stable behavior without drift or undesired oscillations. Since all actuators are supplied through a common manifold, the measured pressure represents the global internal pressure of the actuator array.

#### 3.1.5 Reference pressure determination for controller design

The pneumatic system was characterized by varying the pump input (PWM) and measuring the resulting pressure, force, and current across 15 trials. The operating points corresponding to clinically defined stimulation levels were identified by matching the measured forces with the clinical reference values.

For the light stimulation level, the actuator produced μ=0.640 N ( σ=0.116 N), corresponding to μ=2.132 kPa ( σ=0.741 kPa) and current μ=0.057 A ( σ=0.011 A). For the moderate stimulation level, the actuator produced μ=1.286 N ( σ=0.135 N), corresponding to μ=5.539 kPa ( σ=0.831 kPa) and current μ=0.107 A ( σ=0.010 A).

These results demonstrate that the pneumatic system can reproduce clinically observed force levels while maintaining stable pressure and electrical operating conditions.

##### 3.1.5.1 Pressure-force relationship

**FIGURE 10:**
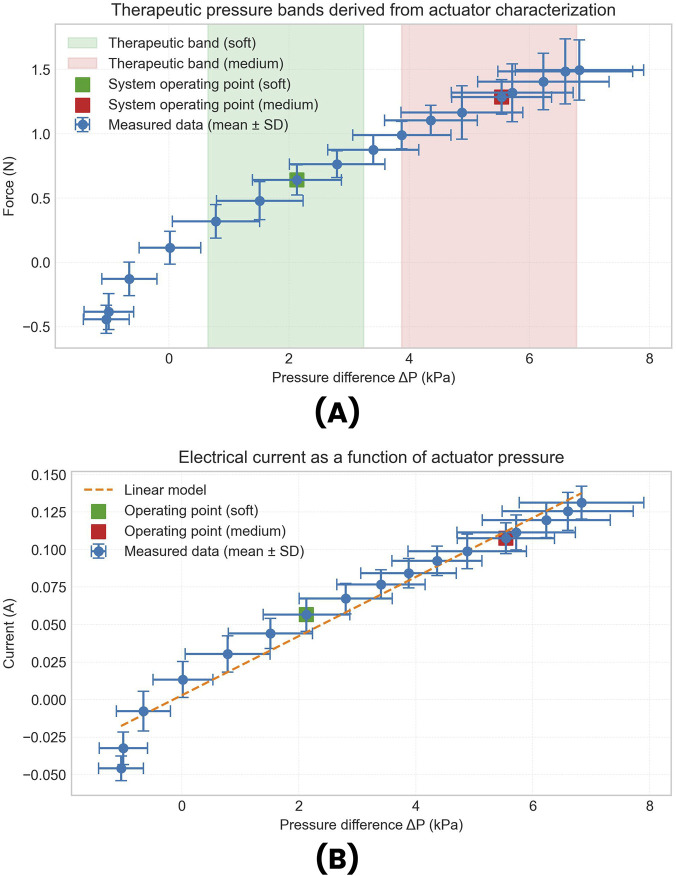
Pneumatic system characterization: **(A)** force generated as a function of pressure difference, with shaded regions indicating therapeutic bands. **(B)** Electrical current consumption as a function of pressure, with linear regression fit.

The relationship between actuator pressure and generated force (Figure 10A) was well approximated by a linear model, as defined in Equation 19:

F=0.2317 ΔP+0.0253 (19)

This near-linear behavior within the operating range enables direct mapping from clinically measured force intervals to corresponding pressure values, providing a practical basis for control reference definition.

It is noted that small negative values of ΔP are observed at low PWM levels. As defined in Equation 13, the pressure difference is computed relative to an offset value measured during initialization. Although this offset is estimated under nominally unpressurized conditions, small residual pressure may remain in the pneumatic lines due to trapped air or incomplete pressure equalization, introducing a slight bias. Combined with sensor noise, this results in small deviations below zero near the zero-pressure condition. These values do not represent physically meaningful negative pressure, but rather measurement uncertainty.

##### 3.1.5.2 Electrical behavior

The relationship between electrical current and actuator pressure (Figure 10B) was also approximated by a linear model, as defined in Equation 20:

I=0.0197 ΔP+0.0028 (20)

The observed linear trend reflects the increasing mechanical load on the pump during actuator inflation. This predictable relationship enables the definition of current thresholds for safe system operation.

Similarly, small negative current values are observed near zero pressure. Since negative current is not physically meaningful in this system, these values confirm the presence of residual offset and measurement noise in the sensing chain. These deviations are more noticeable at low actuation levels, where the signals approach the sensor resolution limits.

##### 3.1.5.3 Therapeutic pressure bands

Therapeutic pressure intervals were obtained by combining clinical force measurements with pneumatic system characterization. The clinically defined force bands were mapped to pressure using the force–pressure model and intersected with experimentally observed pressure variability.

The resulting pressure ranges were:

$$\Delta P\in \left[0.650,3.244\right]\text{ kPa}$$

for light stimulation, and

$$\Delta P\in \left[3.877,6.786\right]\text{ kPa}$$

for moderate stimulation.

These results confirm that actuator pressure provides a reliable proxy for the applied tactile force. The linear relationships between pressure, force, and current enable the use of pressure as the primary control variable, while current is used as a safety constraint.

These experimentally derived models and pressure intervals directly define the reference bands used by the deadband control strategy, establishing a direct link between clinical measurements and embedded control implementation.

### 3.2 Closed-loop control system performance

The performance of the proposed system was evaluated to determine its ability to regulate actuator pressure within clinically defined therapeutic bands under dynamic stimulation conditions. Two control strategies were compared: (1) a pressure deadband controller (Controller 1) and (2) a refined deadband controller incorporating actuation tuning and electrical current protection (Controller 2). This comparison allows quantifying the impact of improving actuation strategy and integrating electrical constraints into the control loop.

Controller 1 was designed to maintain pressure within the therapeutic band derived from clinical characterization. However, under sequential pouch activation, the controller exhibited limited regulation capability, with frequent deviations from the target range, particularly during transitions between actuators.

The relatively low percentage of time within the therapeutic band observed for Controller 1 (30.8%) is primarily attributed to the fast dynamics of the pneumatic system. A sufficiently high PWM value (PWM = 225) is required to ensure that the actuator reaches the therapeutic pressure range within the limited stimulation time $\left(T_{r}=1.739\;s\right)$ . However, this high actuation level results in rapid air injection into the actuator.

Due to the compressibility of air and the dynamics of the pneumatic circuit, there is an inherent delay between the control action and pressure stabilization. Consequently, even after the control signal is deactivated upon reaching the upper bound, pressure continues to increase, producing overshoot. This effect is further amplified by the relatively small internal volume of the actuator, which causes pressure to rise quickly for a given airflow. As a result, the system enters the therapeutic band but exits it rapidly, reducing the effective time within the desired range.

To address these limitations, Controller 2 introduces two modifications: adjustment of PWM levels to better match the dynamic response of the pneumatic system, reducing actuation aggressiveness, particularly in the light stimulation condition, and incorporation of electrical current monitoring as a safety constraint to limit excessive actuation under high load conditions.

**FIGURE 11:**
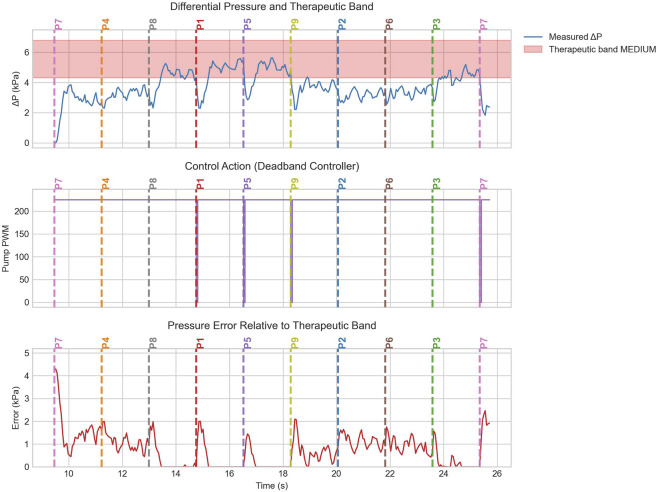
Experimental response using Controller 1 during the diagonal pattern at moderate intensity with a 25 mm spacer. The top panel shows ΔP relative to the therapeutic band, the middle panel shows the PWM control signal, and the bottom panel shows the instantaneous pressure error. Vertical dashed lines indicate pouch transitions.

**FIGURE 12:**
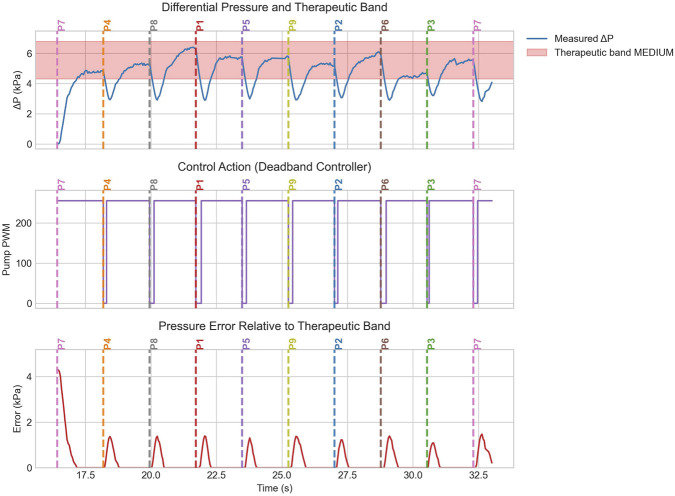
Experimental response using Controller 2 under the same conditions as Figure 11. The pressure trajectory remains closer to the therapeutic band, with reduced error magnitude and faster recovery after pouch transitions.

Figures 11, 12 illustrate representative responses under identical conditions. In Controller 1, pressure deviations are evident following pouch transitions, leading to large error peaks and delayed recovery. In contrast, Controller 2 maintains the pressure trajectory closer to the therapeutic band, with reduced peak errors and improved transient response. This behavior indicates that the refined actuation strategy reduces overshoot, while the current constraint provides an additional bound that enhances robustness during transient conditions.

#### 3.2.1 Overall controller performance

To quantitatively evaluate performance, three metrics were considered: percentage of time within the therapeutic band, mean pressure error, and root-mean-square (RMS) error. Table 1 summarizes the results across all stimulation patterns, intensity levels, and repetitions.

**TABLE 1:** Overall performance comparison between controllers. Values are reported as mean ± standard deviation across all trials.

Controller	Time in band (%)	Mean error (kPa)	RMS error (kPa)
Controller 1 (deadband)	30.8 ± 18.6	0.68 ± 0.31	0.92 ± 0.41
Controller 2 (refined deadband +current constraint)	76.1 ± 6.4	0.15 ± 0.09	0.38 ± 0.19

**TABLE 2:** Robustness evaluation of controller 2 for different spacer thicknesses.

Spacer thickness (mm)	Time in band (%)	Mean error (kPa)	RMS error (kPa)
25	76.3 ± 7.1	0.10 ± 0.03	0.22 ± 0.03
50	81.5 ± 1.9	0.08 ± 0.01	0.20 ± 0.02
17.5	76.0 ± 4.3	0.08 ± 0.02	0.20 ± 0.02

Controller 2 significantly improves pressure regulation performance. The time within the therapeutic band increases from 30.8% to 76.1%, while the mean error is reduced by approximately 78% and the RMS error by approximately 59%. In addition to improved accuracy, Controller 2 exhibits substantially lower variability, indicating more consistent behavior across trials.

These results demonstrate that the combination of actuation tuning and safety-constrained control improves system stability by reducing over-actuation and mitigating transient overshoot.

#### 3.2.2 Robustness to interface thickness

The robustness of Controller 2 was evaluated under different mechanical interface conditions using spacer thicknesses of 25 mm, 50 mm, and 17.5 mm, which emulate variations in actuator–surface interaction, as summarized in Table 2.

The controller maintains stable performance across all tested configurations. The time within the therapeutic band remains consistently high, and the error metrics show minimal variation despite changes in mechanical coupling. This indicates that the control strategy is robust to variations in interface conditions, which are expected in practical applications.

Overall, these results demonstrate that the proposed control approach achieves reliable pressure regulation within clinically defined limits, while maintaining robustness and incorporating electrical safety constraints.

## 4 Discussion

The results demonstrate that clinically inspired tactile stimulation can be quantitatively reproduced using a soft pneumatic robotic system, with controlled force levels, spatial activation, and temporal consistency. By integrating clinical characterization, pneumatic modeling, and closed-loop control, the proposed framework enables the translation of manual therapeutic interactions into reproducible robotic actuation.

The experimental characterization of tactile forces revealed that neonatal stimulation operates within a narrow sub-Newton range, with mean values of 0.594 N (light) and 1.267 N (moderate). The relatively low variability observed in both conditions supports the use of statistically defined bounds as reliable control references. These findings highlight the importance of grounding robotic actuation parameters in clinically measured data, rather than relying solely on actuator capabilities.

The pneumatic characterization established approximately linear relationships between pressure, force, and current within the operating range. In particular, the force–pressure relationship (F=0.2317 ΔP+0.0253) enables direct mapping of clinically defined force intervals into pressure-based control targets. Similarly, the current–pressure relationship (I=0.0197 ΔP+0.0028) provides a predictable representation of the electrical load associated with actuator inflation. These linear models simplify the control problem and support the use of pressure as the primary regulated variable, while enabling current-based safety constraints.

The evaluation of the control strategies highlights the importance of properly matching actuation dynamics to the physical behavior of the pneumatic system. The baseline deadband controller achieved limited regulation performance, maintaining the pressure within the therapeutic band only 30.8% of the time and exhibiting a mean error of 0.68 kPa. These deviations are primarily associated with transient dynamics introduced by sequential actuator activation, combined with aggressive actuation that leads to overshoot.

In contrast, the refined controller significantly improves performance, increasing the time within the therapeutic band to 76.1%, while reducing the mean pressure error to 0.15 kPa and the RMS error to 0.38 kPa. This corresponds to reductions of approximately 78% and 59%, respectively. The improvement is primarily attributed to the adjustment of PWM levels, which reduces actuation aggressiveness and better aligns the control action with the system dynamics.

The inclusion of current-based protection introduces an additional constraint that bounds the actuation process. Although the current threshold is not continuously activated under all operating conditions, it provides a safety mechanism that prevents excessive actuation under higher load scenarios. This contributes to improved robustness of the control system without interfering with nominal operation.

From an implementation perspective, additional design considerations were incorporated to improve measurement reliability and system stability. A pressure relief mechanism was included in the pneumatic circuit to mitigate the effects of trapped air within the tubing and actuator network. Residual air can introduce pressure offsets and transient artifacts, affecting both measurement accuracy and control performance. The inclusion of a relief path allows the system to release excess pressure and stabilize baseline conditions prior to operation.

In parallel, a custom printed circuit board (PCB) was developed to organize the electrical connections of sensors, valves, and the pump. This integration reduces wiring complexity, improves signal integrity, and enhances repeatability by minimizing noise and connection variability. These hardware-level refinements contribute to more stable operation and more reliable experimental measurements, particularly in pneumatic systems with fast actuation cycles where small inconsistencies can affect transient behavior.

The robustness analysis further confirms the stability of the control strategy under varying mechanical interface conditions. Across spacer configurations of 25 mm, 50 mm, and 17.5 mm, the controller maintained time-in-band values between 76.0% and 81.5%, with minimal variation in error metrics. This behavior suggests that the proposed approach is relatively insensitive to moderate changes in actuator–surface coupling, which is a critical requirement for wearable biomedical devices.

From a system-level perspective, the combination of clinically derived reference values, experimentally validated pneumatic models, and safety-oriented control design enables a consistent mapping from clinical input to robotic output. The use of a multi-actuator matrix allows the reproduction of spatial stimulation patterns, while the control system ensures that the applied stimulus remains within predefined therapeutic limits under dynamic conditions.

Despite these contributions, several limitations should be considered. First, the characterization of tactile forces was conducted using measurements from a single neonatology specialist under controlled experimental conditions. As a result, the derived force ranges reflect the practice of an individual operator and should not be interpreted as representative of the full variability present in clinical settings. Inter-operator differences in applied force, technique, and patient-specific factors may lead to variations that are not captured in this study. Second, the experiments were performed using a neonatal mannequin, which does not fully replicate the mechanical properties of human tissue. Finally, the control strategy regulates pressure rather than directly measuring contact force, which may introduce discrepancies in force transmission.

## 5 Conclusion

This work presented the design and experimental validation of a soft pneumatic robotic system capable of reproducing clinically inspired tactile stimulation for neonatal therapy. The proposed approach integrates clinical force characterization, pneumatic system modeling, and closed-loop control within a unified framework.

A central contribution of this study is the establishment of a direct mapping between clinically measured tactile forces and pneumatic control variables. By experimentally identifying linear relationships between force, pressure, and current, the system enables the definition of pressure-based control references that are both clinically grounded and technically implementable.

The evaluation of control strategies demonstrated that appropriate tuning of actuation parameters is critical for achieving stable pressure regulation in pneumatic systems. The refined controller achieved a time within the therapeutic band of up to 76.1%, while substantially reducing pressure error and variability compared to a baseline deadband approach. These results highlight the importance of matching actuation dynamics to system behavior in order to mitigate overshoot and improve regulation performance.

The incorporation of electrical current monitoring provides an additional safety constraint that bounds the actuation process under high-load conditions. Although this constraint is not continuously active under all operating regimes, it enhances system robustness and ensures safe operation without compromising control performance.

The system further demonstrated robustness to variations in mechanical interface conditions, maintaining stable performance across different spacer configurations. This characteristic is essential for practical deployment in wearable biomedical applications, where consistent mechanical coupling cannot be guaranteed.

Overall, the results confirm that soft pneumatic actuation can be used to reproduce clinically derived tactile stimulation patterns in a controlled, safe, and repeatable manner. By bridging clinical observation and robotic implementation, this work contributes to the development of quantitative and reproducible tactile stimulation technologies for neonatal care.

Future work will focus on incorporating distributed force sensing for direct feedback, increasing actuator density to improve spatial resolution, and validating the system in clinical environments involving neonatal patients.

## Data Availability

The datasets presented in this study can be found in online repositories. The names of the repository/repositories and accession number(s) can be found in the article/Supplementary Material.

## Nomenclature

F: Applied force (N)
I: Electrical current (A)
ΔP: Pressure difference (kPa)
P: Measured pressure (kPa)
$P_{\mathrm{offset}}$: Pressure offset obtained during initialization (kPa)
V: Sensor output voltage (V)
u(t): Control signal at time t
PWM: Pulse width modulation duty cycle (%)
ADC: Analog-to-digital converter reading
$I_{\mathrm{fuse}}$: Current threshold for protection (A)
$T_{r}$: Inflation time per actuator (s)
$T_{\mathrm{pattern}}$: Duration of a stimulation pattern (s)
$T_{\mathrm{therapy}}$: Total therapy duration (s)
μ: Mean value
σ: Standard deviation
a,b: Linear model coefficients (current estimation)
