# Supplementary figures and images for: Design and experimental validation of a soft pneumatic robotic device for preterm infant skin-to-skin tactile therapy
Source: Front Robot AI. 2026 May 28;13:1839026. doi: 10.3389/frobt.2026.1839026 (PMC13253241; doi:10.3389/frobt.2026.1839026)

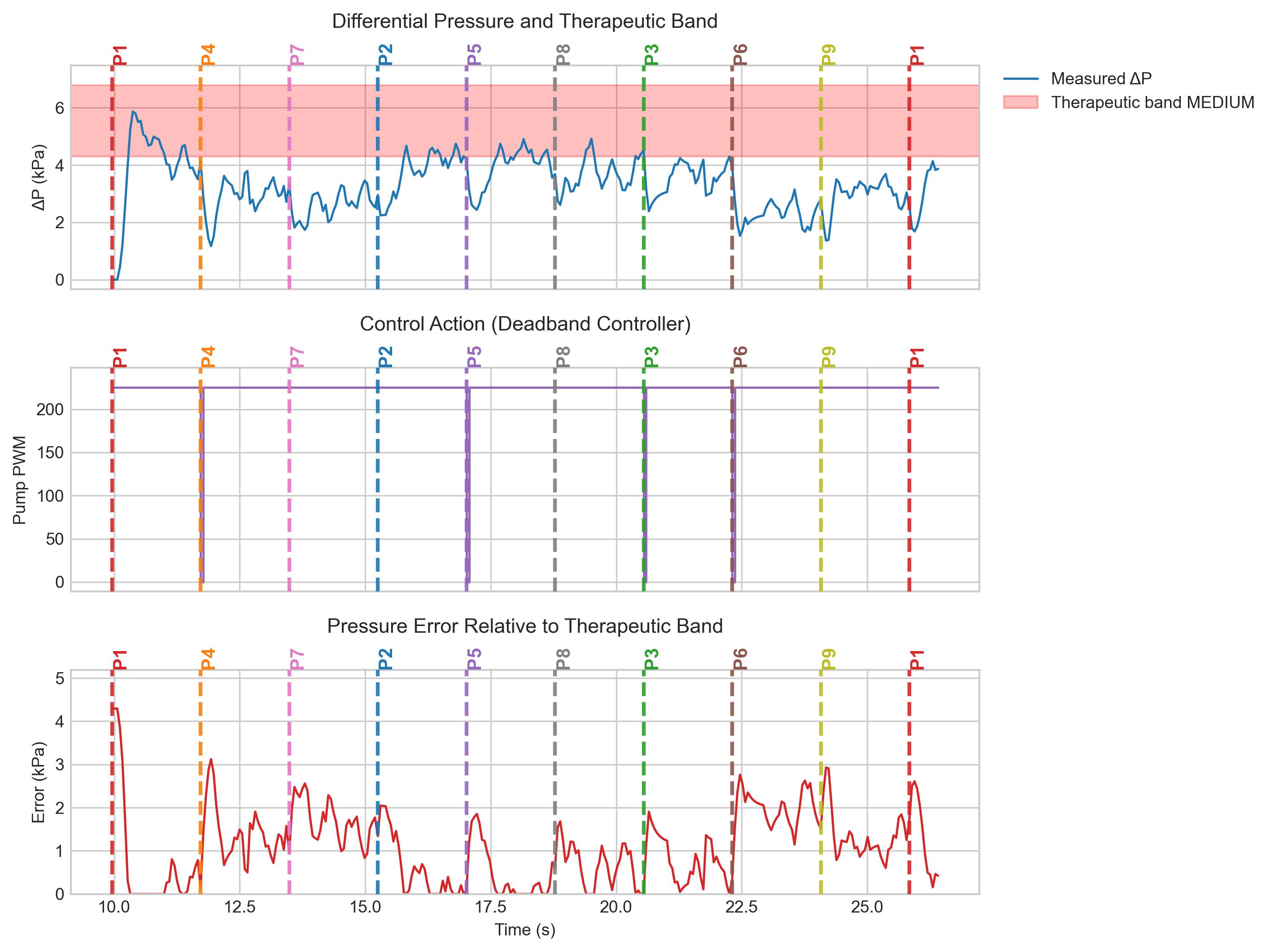

Supplement: Supplementary file 1 [file Supplementaryfile2.zip › Figures/supplementary/control1_extra_patternA_medium_25mm.jpg]

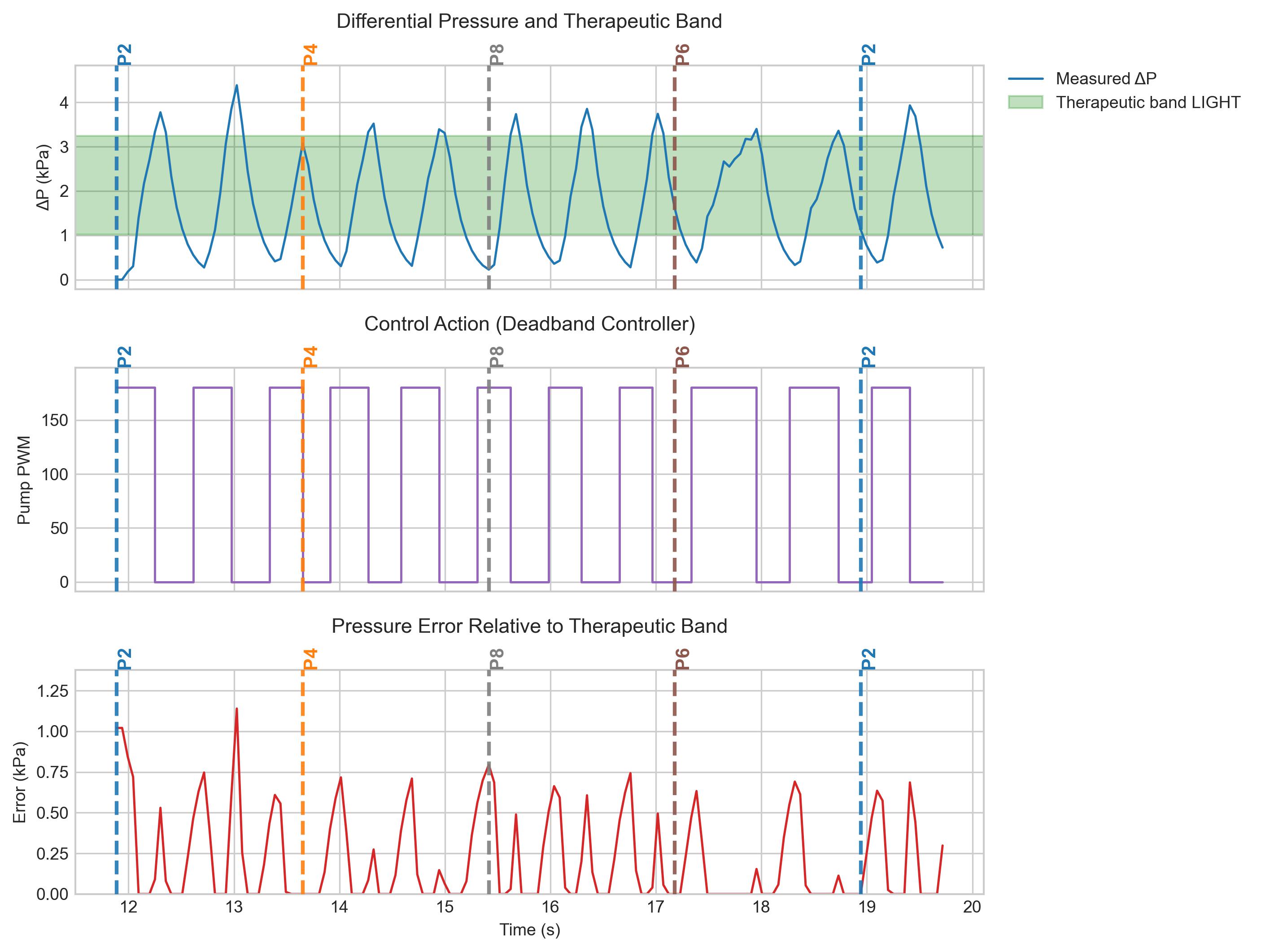

Supplement: Supplementary file 1 [file Supplementaryfile2.zip › Figures/supplementary/control1_extra_patternB_light_25mm.jpg]

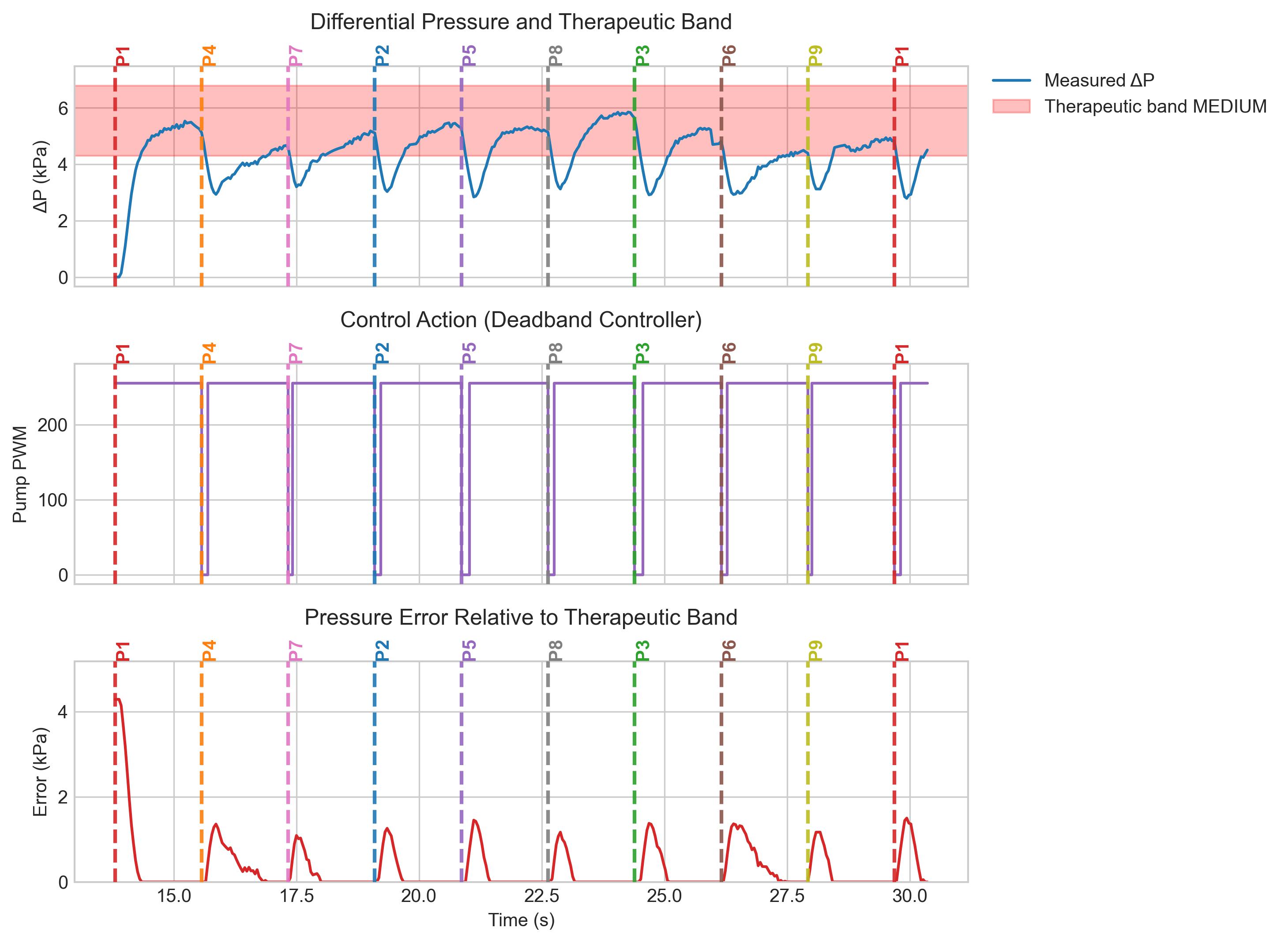

Supplement: Supplementary file 1 [file Supplementaryfile2.zip › Figures/supplementary/control2_extra_patternA_medium_17mm.jpg]

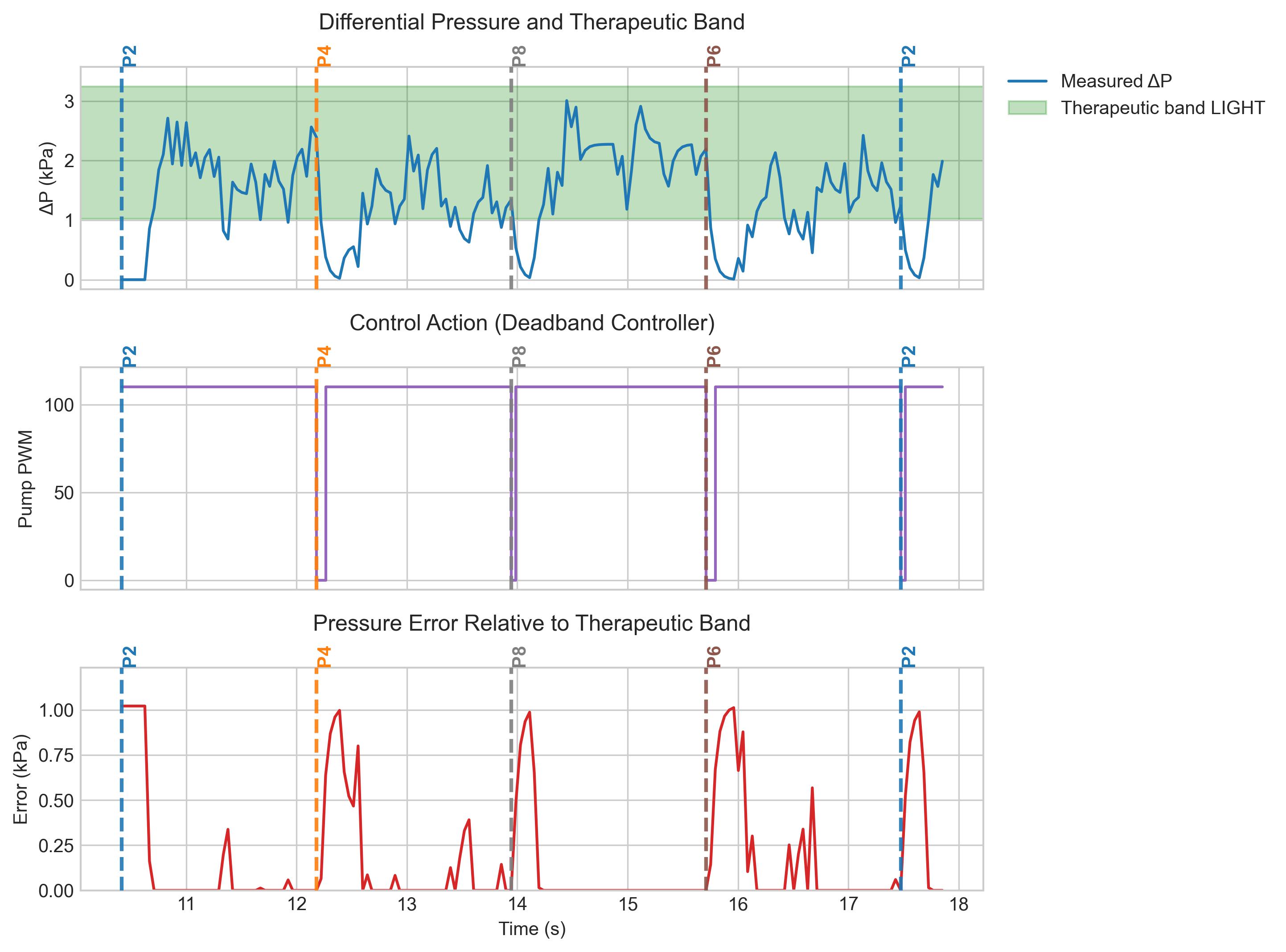

Supplement: Supplementary file 1 [file Supplementaryfile2.zip › Figures/supplementary/control2_extra_patternB_light_25mm.jpg]

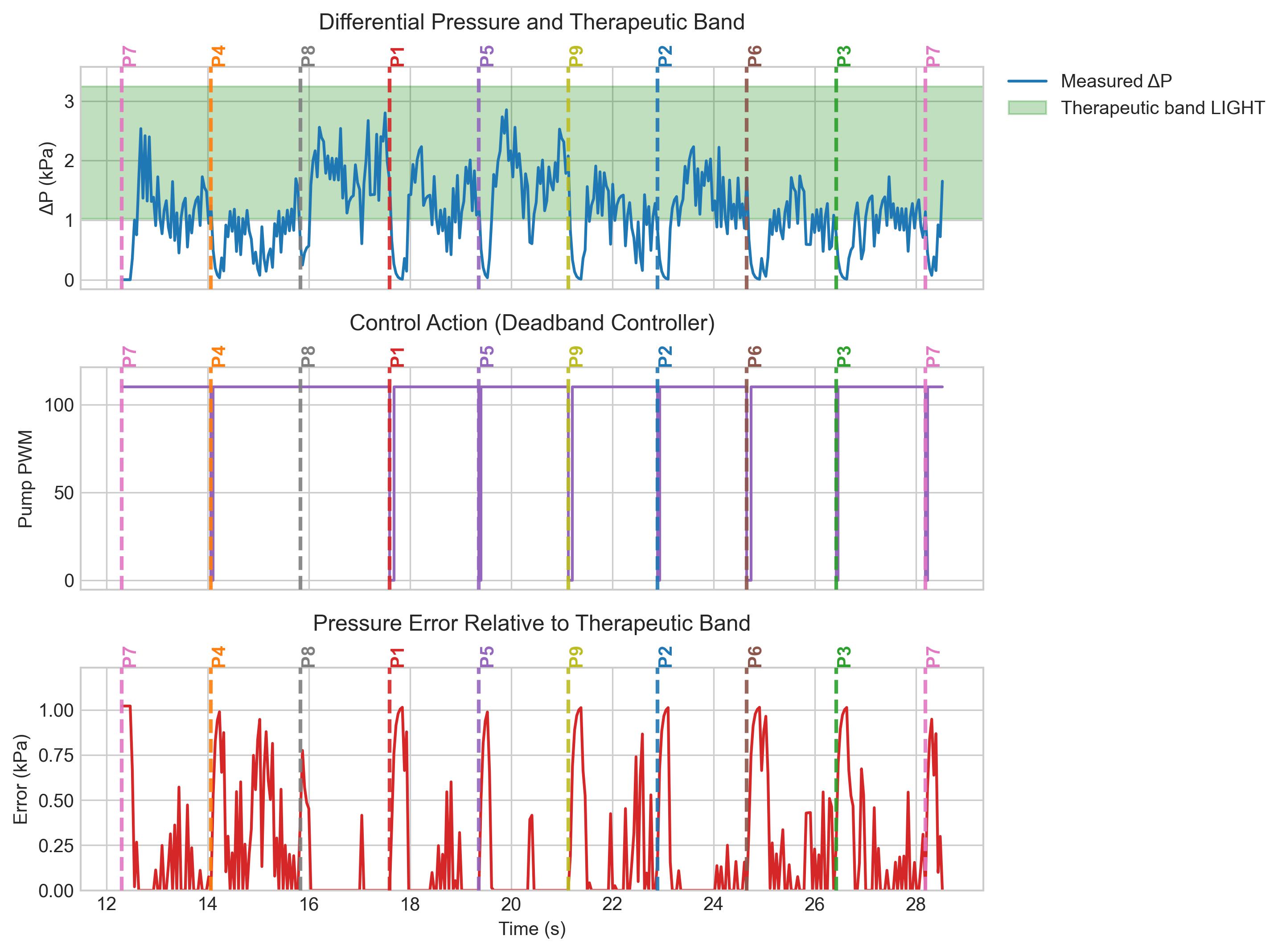

Supplement: Supplementary file 1 [file Supplementaryfile2.zip › Figures/supplementary/control2_extra_patternC_light_17mm.jpg]

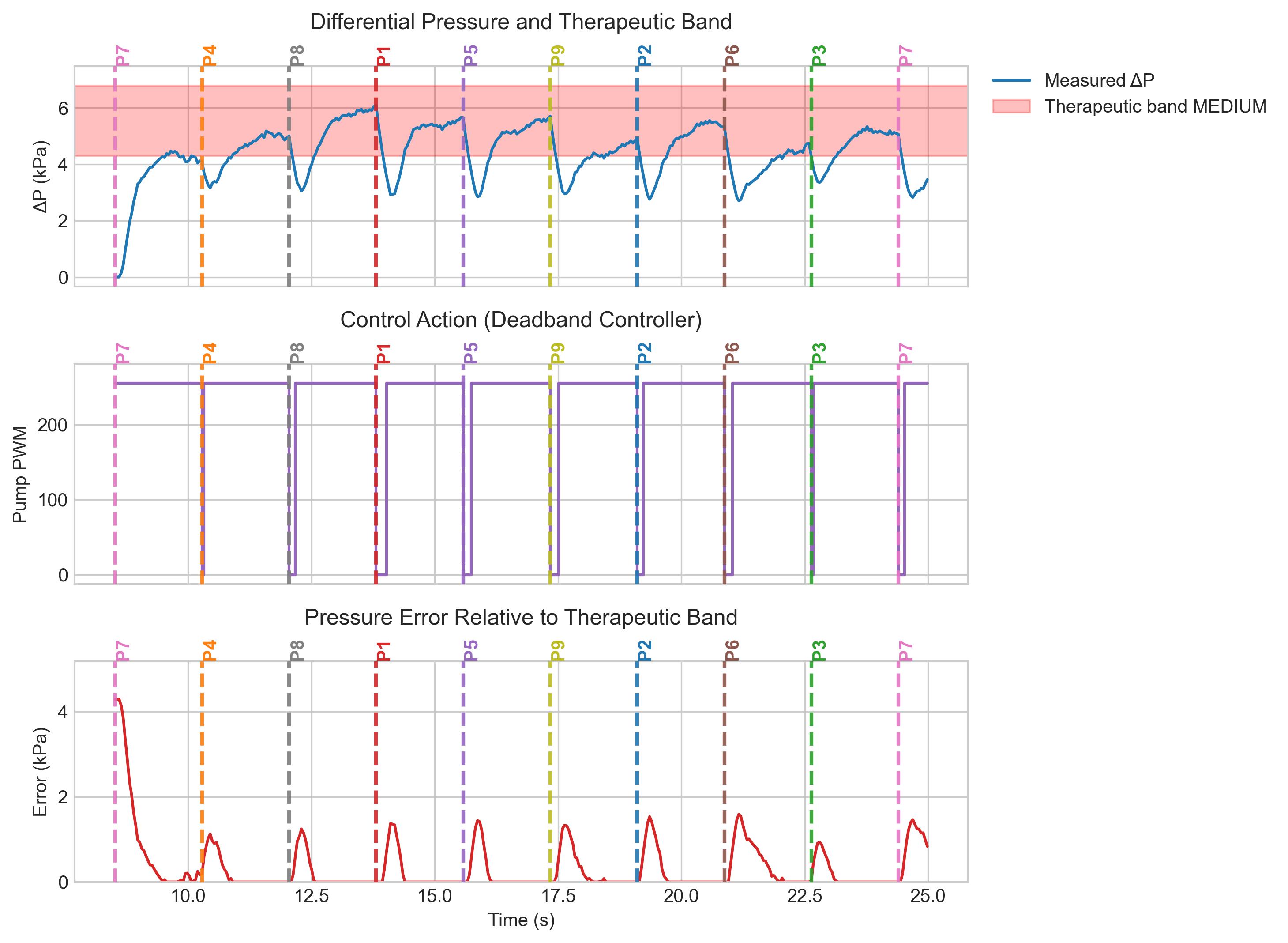

Supplement: Supplementary file 1 [file Supplementaryfile2.zip › Figures/supplementary/control2_extra_patternC_medium_50mm.jpg]
